# Lying Behaviour in Dairy Goats: Effects of a New Automated Feeding System Assessed by Accelerometer Technology

**DOI:** 10.3390/ani11082370

**Published:** 2021-08-11

**Authors:** Ines Maurmann, Bianca A. E. Greiner, Stanislaus von Korn, Maren Bernau

**Affiliations:** 1 Fakultät Agrarwirtschaft, Volkswirtschaft und Management, Hochschule für Wirtschaft und Umwelt Nürtingen-Geislingen, Neckarsteige 6–10, 72622 Nürtingen, Germany; 2 Institut für Angewandte Agrarforschung, Hochschule für Wirtschaft und Umwelt Nürtingen-Geislingen, Hechinger Straße 12, 72622 Nürtingen, Germany; bianca.greiner@hfwu.de (B.A.E.G.); stanislaus.korn@hfwu.de (S.v.K.)

**Keywords:** dairy goat, lying behaviour, automated feeding system, accelerometer technology

## Abstract

**Simple Summary:**

Goat farming is becoming more important in Germany and as dehorning is forbidden, it is necessary to facilitate animal welfare among horned and mixed-horned herds. In this study an optimized automatic concentrated feeding system was installed in a mixed-horned herd and lying behaviour was detected by accelerometer technology. Results show a seasonal progression of lying behaviour in dairy goats and an adjustment of behavioural differences between horned and hornless goats with the new feeding system.

**Abstract:**

The aim of this study was to evaluate lying behaviour in dairy goats before and after installation of an optimized automatic concentrated feeding system (AFS). A mixed-horned herd of Bunte Deutsche Edelziege was used. As many agonistic interactions between goats happen at the feeding place, a new automated feeding system was installed to better fulfil the needs of horned goats. Lying behaviour is an indicator to ascertain animal welfare of ruminants. In order to measure lying behaviour accelerometer technology was used and verified by video analyses. The results show an agreement of 99.62–99.93% per lying time by comparing accelerometers to video data. Over all goats, a mean ± SD lying time (LT) of 11.78 ± 1.47 h/d, a mean ± SD lying bout duration (LBD) of 0.51 ± 0.10 h/bout and a mean ± SD frequency of lying bouts (FLB) of 24.35 ± 5.57 were found. Lying behaviour follows a seasonal progression with significant lowest LBD and highest FLB in summer. With the old AFS significant differences in LBD and FLB were detected between horned and hornless goats, but with the new AFS results were adjusted. Findings suggest that changes in feeding management do not affect the general seasonal progression of lying behaviour but can affect the behavioural differences between horned and hornless dairy goats.

## 1. Introduction

In Germany, particularly in federal state Baden-Württemberg, dairy goat farming is becoming increasingly important as herd sizes are rising [1,2]. The breeds most strongly represented in Germany are Bunte and Weiße Deutsche Edelziege [3]. These breeds are often horned, which is of major interest for housing and feeding and results in special needs, since dehorning is forbidden in Germany [4].

Due to their species-specific behaviour, agonistic interactions and horn-induced injuries are often found, especially as a result of competition during feed intake. Limited resources such as those for feeding can be a reason for aggressive behaviour in goats [5]. With the increasing number of goats for one feeding place the frequency of aggressive interactions between goats increases [6]. By comparing horned and hornless goats, horned goats have a higher need for individual space at the feeding place than hornless goats [7,8,9], which results in more feeding places and specific needs in mixed-horned herds. Injuries affect animal well-being, animal health, and result in economic losses [10].

Additionally, individual feed quantities adapted to a corresponding milk yield are of great interest in dairy goats. Individual feeding results in higher feed efficacy, due to individual preferred feeding patterns of goats [11]. In calf-management automated feeding systems are used to adjust individual needs and to monitor feeding behaviour [12]. In order to increase feed efficiency in dairy goats concentrated feeding stations are needed in dairy goats as well. Adapted AFS may lower the risk of severe injuries due to aggressive behaviour during feeding. However, there is a lack of concentrated feeding stations, which are sufficient for horned dairy goats without leading to aggressive behaviour and injuries. Most AFS were built for calves or hornless goats, but especially for hornless goats some special aspects are already known. Goats prefer elevated feeding platforms [13]. Additionally, (head) partitions influence the feeding and social behaviour of goats positively [14]. Barriers between feeding places result in less agonistic behaviour in the feed barrier and in the total feeding area [15]. High-ranked/dominant goats tend to monopolize the space in front of a feeding place by lying in front of them [6]. Therefore, automated feeding systems need to be constructed to reduce agonistic behaviour in the feeding area and avoid monopolism of high-ranked animals but allow individual feeding. 

For the evaluation of a feeding system, besides functional tests, behavioural measurements can be used in order to evaluate the effect on animal health and welfare before and after installation. Several factors can be defined. One aspect especially important for ruminants is lying behaviour [16]. Traditional animal monitoring methods are labour intensive, human observation of animals. Accelerometer technology offers the possibility to remotely monitor animal behaviour non-invasively and continuously for 24 h a day and 7 days a week. This technique has been used successfully in goats and other farm animals (calves: [12], cattle: [17,18], goats: [19]).

The present study evaluated a new feeding system, specially built for horned and hornless goats with respect to their natural behaviour patterns. With accelerometer technology, lying times were studied before and after installation of the new system. The aim of this study was to answer the question whether the new feeding system influences the lying time in dairy goats, with special attention to horned and hornless goats. 

## 2. Materials and Methods

### 2.1. Animals, Housing and Management

A dairy goat herd of Bunte Deutsche Edelziege was used for this study. Goats were kept in a mixed group with horned and hornless dairy goats (mean ± SD: 67.5 ± 4.09 animals). The measurements were performed from April 2019 until October 2020 (see Table 1). Meanwhile, the automatic concentrated feeding system (AFS) was changed, while management, hay feeding and pasture access were kept similar.

**Figure 1:**
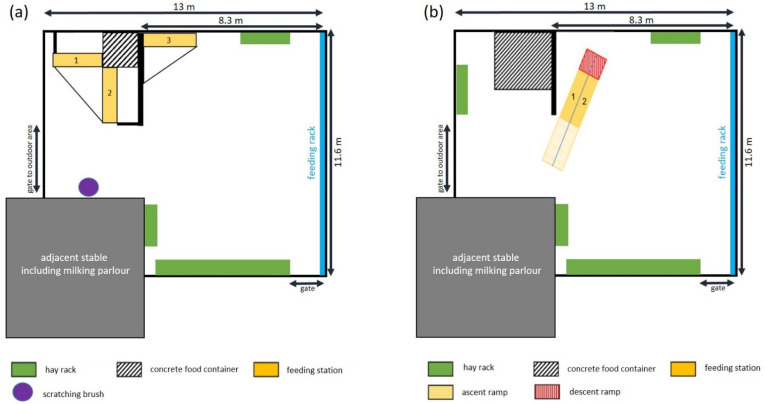
Schematic representation of the stable before (**a**) and after (**b**) installation of the new automatic feeding system in bird’s eye view. (Numbers 1, 2 (and 3) show the different feeding stations. “m” = meters).

**Figure 2:**
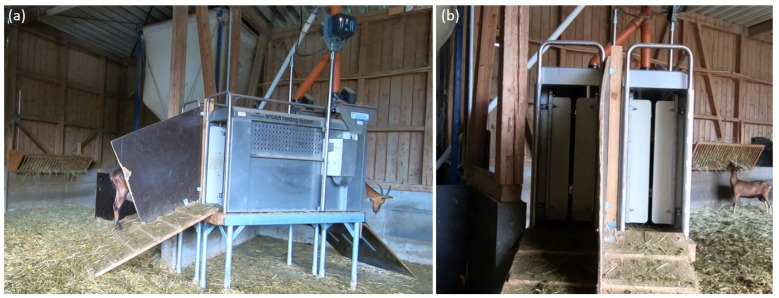
Photographs of the new optimized automatic feeding system. (**a**) The station in side view. (**b**) The divided entrance with the ramp.

Goats were housed in an outdoor climate barn with around 135 m^2^ ground area. The herd was supplied with concentrated feed by an AFS and additionally during milking. This system consisted of three individual feeding stations, positioned around the concrete food container (see Figure 1a), which reduced the space to 123 m^2^. These stations were built with a backward exit. After 10 October 2019, the three old stations were replaced by two optimized ones (Fa. Hanskamp and Dedden). The new AFS was adapted to goat-specific behaviour and was constructed as a walk-through, built on an elevated platform (see Figure 2; for more information see Greiner et al. [20]). After that day, goats had a space of 129.5 m^2^, due to the different positioning compared to the former AFS (see Figure 1b). On Wednesday 30 October 2019, a partition between the two stations of the new AFS on the entrance-platform was installed (see Figure 2b). An additional hay rack (see Figure 1b and Figure 2b) was installed after the measurement period of October 2019.

Goats were milked twice a day in a milking parlour, starting between 7:30 a.m. and 10:00 a.m. in the morning and between 6:30 p.m. and 8:00 p.m. in the evening. Mean annual milk yield evaluation of all goats in the herd received 751.42 kg milk/goat (255.96 milking days) in 2019 and 766.23 kg milk/goat (258.40 milking days) in 2020. Goats have an average life performance of over 3.000 kg milk (3.607 kg milk in 2019 and 3.654 kg milk in 2020). The farmer can program AFS in order to offer concentrated food according to the individual milk yield of each goat. In the present study, the farmer programmed a start with 0.1 kg/d in late lactation, followed by a slow increase until the highest needs after kidding (fixed at about 0.8 kg/d for this herd). AFS calculates the number of daily feedings individually according to the amount of concentrated feed and the number of visits of each goat. Each feeding contained a maximum of 0.2 kg. Hay was given four times a day, with additional grass and alfalfa in summer. In summer, depending on the weather conditions, goats had different grazing times on pasture. Access to water was given ad libitum.

### 2.2. Measurements

Measurements took place from April 2019 until October 2020, with 3 measurement periods per lactation period (start–middle–end of lactation; see Table 1), including two periods of kidding from January to March (2019 and 2020). For activity detection, 20 accelerometers (MSR145WD, MSR Electronics GmbH) were used. In each measurement period 20 goats were randomly chosen (in consideration of their horn status) and the accelerometers were fixed at the hind leg with elastic tape. Two of these 20 goats (one horned and one hornless goat) were used in each measurement period. Accelerometers were set with a 5 s interval. For data analysis a 10 s interval was used to remove false readings. Accelerometers were started at 0:00 each measurement period and detected seven days (only in April 2019 measurements started at 12:00).

Additionally, during the measurements, four cameras were installed in the stable (Mobotix S15D, Mobotix AG, Langmeil, Germany) to record animal behaviour during day and night. In order to validate accelerometer data, video data were analysed for one randomly chosen goat, which could be identified consistently. For this goat, times of lying down and getting up were analysed and compared with the accelerometer data of that day by manual detection using MxManagementCenter (Version 2.4.3, Mobotix AG, Langmeil, Germany).

Ethical approval for the study was obtained from the University of Applied Sciences Nürtingen-Geislingen Ethics Committee (Ref: 2019_01) and was in accordance with local and national guidelines [4,21]. 

### 2.3. Statistical Analyses

All animals equipped with accelerometers were analysed statistically. For statistical analyses the following response variables were created:

- Lying time (h/d per goat) (LT), representing means of all daily lying times of all goats.
- Lying bout duration (h/bout) (LBD), representing (the mean) daily lying bout duration with start and end point of all goats.
- Frequency of lying bouts (n/d and goat) (FLB), representing means of all daily lying bouts of all goats.

The statistical software package R Studio (The R Foundation for Statistical Computing, Zurich, Switzerland) was used for a three-factor variance analysis (three-way-ANOVA) procedure using a linear model and performing paired Tukey’s *t*-tests with the emmeans function. The three explanatory variables “month”, “year” and “horn status” were treated as fixed effects and interactions between all of them were assumed (two-way interactions and three-way interaction). Missing values have been removed for statistical analysis. The significance level was set at *p* < 0.05.

## 3. Results

### 3.1. Video Data

Comparing lying times of the accelerometer measurements with video data (of one random goat) reached an agreement of 99.62–99.93% per lying time.

### 3.2. Accelerometer Data

Over all goats, a mean ± SD LT of 11.78 ± 1.47 h/d, a mean ± SD LBD of 0.51 ± 0.10 h/bout and a mean ± SD FLB of 24.35 ± 5.57 were found.

**Figure 3:**
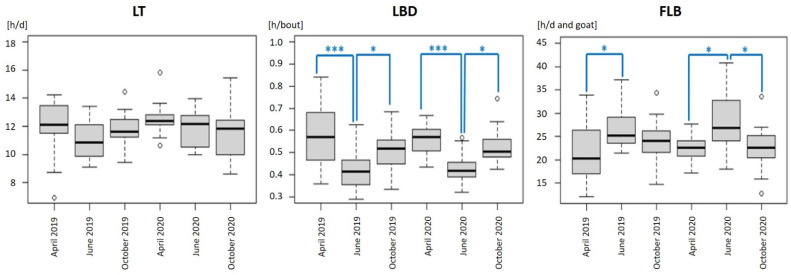
Boxplot diagrams representing the mean duration of lying time (LT), the mean lying bout duration (LBD) and the mean frequency of lying bouts (FLB) for all examined goats. *** representing *p* ≤ 0.0001; * representing *p* ≤ 0.05.

Comparing data of April to June to October, goats showed the same tendency in lying bouts (Figure 3): (a) from April to June, goats showed a highly significant decrease in LBD with a simultaneous significant increase in FLB (both in 2019 and 2020), and (b) from June to October goats showed a significant increase in LBD (2019 and 2020) with a simultaneous decrease in FLB (significant in 2020).

Additionally, comparing horned and hornless goats, significant differences were detected in 2019 (see Table 2). Horned goats had a significantly higher FLB than hornless goats (April and June 2019), whereas hornless goats had significantly longer LBD (April and June 2019). In 2019, horned goats had shorter LBD but higher FLB than hornless goats. No differences were observed for LT, neither in 2019 nor in 2020. In 2020, no differences were detected regarding LBD and FLB between horned and hornless goats.

Comparing the seasonal progression divided for horned and hornless goats, similarities were observed (Table 2). Hornless and horned goats showed the same seasonal progression with highest FLB and shortest LBD in June.

## 4. Discussion

### 4.1. Accelerometer Technique

Comparison of accelerometer and video data showed comparable results. Therefore, as shown in goats [19] and other animals [12,17], this technique results in reliable data regarding lying behaviour in goats without the need for labour intensive video analyses.

Observation of video recordings in the present study showed scratching behaviour with the hind legs for a duration between 5 and 8 s. Therefore, data were evaluated with a 10 s interval, although accelerometers were set to a 5 s interval for recording. The results of the performed random video analyses (99.62–99.93% agreement of lying) support the possibility of using accelerometers with a 10 s interval for observing lying behaviour in ruminants. This is confirmed by Robert et al. [17], who found a total agreement of 99.2% in lying behaviour of cattle with intervals of 3, 5 and 10 s.

In the present study, LBD was 0.51 ± 0.10 h/bout (mean ± SD) and FLB was 24.35 ± 5.57 bouts (mean ± SD); therefore, goats were more active than in the study of Zobel et al. [19], who documented an LBD of 1.2 h/bout and a FLB of 12 lying bouts. This supports using a shorter measuring interval than the 1 min interval of Zobel et al. [19], although they likewise found a sensitivity of 99.7% and a specificity of 99.5% in comparing video and accelerometer data of lying behaviour of goats. Additional analyses should be performed to evaluate scratching behaviour and changes in lying behaviour in more detail, in order to gain more information about ideal recording intervals in goats, especially in different breeds.

### 4.2. Lying Parameters Comparatively Shown for Old and New AFS

Comparing lying parameters in two following years gives insight into the activity pattern of dairy goats. Significant differences were detected during the lactation cycle in both years. These differences were observed for the former and the newly installed optimized AFS. However, differences between horned and hornless goats were only evaluated with the former AFS. Comparing the different lying parameters (LT, LBD, FLB) assessed from all dairy goats, no difference could be detected comparing 2019 and 2020; therefore, no differences were found which can be related back to the new AFS. Therefore, in the present study no direct conclusion can be drawn whether the new AFS influences lying parameters of dairy goats.

In 2020, LT from June to October decreased, in contrast to 2019. This might be explained by the fact that in October 2019 the new AFS was installed, and with this lying behaviour should be interpreted carefully. Several reconstructions of the AFS happened at the time when the accelerometers were put on, such as the installation of the partition on the entrance-platform and the steepening of the exit-platform. These interruptions may have affected lying times in October 2019.

Significant differences regarding lying behaviour (LBD and FLB) between horned and hornless goats were detected with the old AFS but not with the new AFS. If this adjustment in lying behaviour between horned and hornless goats in a mixed-horned herd is a reason for reduced stress in lactating goats, the changed lying times could have an effect on health, performance and welfare of the dairy goats, as has already been proven in other ruminants such as dairy cows [16]. This must be evaluated with performance data and by video analyses, and additionally by evaluating the behaviour in front of the feeding system. Further studies or evaluation of video data must be examined in order to detect behavioural patterns in more detail. Lying activities seem to be a good variable to evaluate herd activities, and accelerometers on hind legs seem to be an easy-to-use method. Additionally, more focus on horned and hornless goats and their interactions at the automated feeding system is necessary to fully ascertain the effect of the optimized AFS in a mixed-horned herd.

#### 4.2.1. Evaluated Lying Times Compared to Other Studies 

In the present study, an overall LT of 11.78 ± 1.47 h/d (mean ± SD) was examined. This is lower than in studies of Zobel et al. [19,22], who found a mean lying time of healthy goats of 15.45 h/d two to 12 days before and after kidding [22] and 14.5 h/d in late gestation goats [19]. In a study of Patt et al. [23], the mean LT of non-lactating horned goats was 13.22 h/d (in groups of seven goats/group). Additionally, LBD in the study of Zobel et al. [19] was about 1.2 h/bout, but about 50% of the lying bouts in their results were shorter than 30 min. For dairy goats in the present study, examination results were lower. Maybe the differences to Zobel et al. [19,22] are based on a different herd structure, different housing conditions or breed differences, as in their studies Saanen and Alpine crossbred goats in a group of 30 [19] and crossbred goats with mainly Saanen, Alpine and La Mancha in groups of about 42 goats per farm [22] were used. There is a lack of research about several behaviour patterns (e.g., lying and social behaviour) of different goat breeds, so no prediction of behavioural differences between Bunte Deutsche Edelziege and other breeds can be made. Hence, a comparison of lying times of various studies with different housing, management and breeds is difficult, as differences such as group size, horn status and lactation status could influence lying times. Tucker et al. [16] showed that management and housing systems have a great influence on lying times of dairy cows. It is possible that this is adaptable for dairy goats too and could explain the differences in lying times, as management and housing conditions were not the same. 

#### 4.2.2. Seasonal Progression of Lying Times

Focusing on the seasonal progression in a herd of horned and hornless dairy goats, lying periods follow a seasonal progression with significantly lowest LBD in summer (June) and simultaneous significantly higher FLB (Figure 3). These significant differences were observed in both years; therefore, the AFS did not influence this general annual progression. This progression may be a result of the reproductive cycles, the milk yield, the pasture management including grazing times and mosquito plagues or temperature and the climate. Cows also lie down less in summer when temperature increases [24,25].

As the focus of the present study was to identify differences before and after installation of the AFS, this result cannot be explained in total by this study. However, as one example, grazing times were not documented during the study and may be different between both years. This general seasonal progression may not be influenced by small changes in management. Some studies discuss bedding material, such as that of Bøe et al. [26], with straw as the least favourite material for lying (compared to expanded metal, solid wood and mattresses) in dairy goats, especially in moderate temperatures. The stable in the present study uses straw bedding; therefore, this might be a reason for shorter LT in June, but this cannot be confirmed, as stable temperatures were not assessed. One other reason for more interruption in lying times in June could be a higher stocking density indoors during the day, because the floor surface of the outside area becomes very hot during summer [27] and likewise also in indoors areas, which are heated by the sun. As the lying area decreases, lying times decrease too [8,28].

#### 4.2.3. Effect of Horn Status on Lying Times

The study was conducted in a mixed-horned herd. Therefore, differences between horned and hornless goats should be analysed as well. The study design was set up in such a way that the ratio of horned and hornless goats equipped with an accelerometer was adjusted to the ratio in the total herd. Due to the changed ratio over the course of the studies, more horned goats were equipped with accelerometers than hornless goats, especially in 2020 (see Table 1). This effect could have an impact on the data, although the design was chosen to match the total herd.

Significant differences were observed by comparing lying parameters of horned and hornless goats in the present mixed herd. In 2019, horned goats showed significantly higher FLB with simultaneous shorter LBD compared to hornless goats. These differences were only detected in April and June 2019 with the former AFS, but not in October 2019 and 2020. In 2020, no differences were observed between horned and hornless goats regarding LBD and FLB. There was no significant decrease in LBD and no significant increase in FLB of hornless goats from 2019 to 2020 and also no significant increase in LBD and no significant decrease in FLB of horned goats from 2019 to 2020. Nevertheless, the non-significant changes in lying behaviour of horned and hornless goats have converged these two parameters. This might show an adjustment in lying behaviour of horned and hornless goats after the new AFS was installed.

As suggested by previous studies [7,8,9], horn status could have an effect on lying behaviour in mixed-horned herds as needs of horned goats are different than those of hornless goats. These differences in horned and hornless goats may be explained by agonistic or territorial behaviour. This might be confirmed by the fact that these differences were not detected since October 2019, when the AFS was changed to an optimized AFS. 

The new AFS was placed differently than the one before (Figure 1), and with this it offers slightly more space and a differently structured stable. By removing the three old AFS more undisturbed resting areas were created (Figure 1). Especially, more undisturbed space next to walls was created, which fulfills the preference of goats to rest against walls [28]. In addition, after the measurement period of October 2019 a new hay rack was installed which led to more feeding places. The impact on lying behaviour of slightly extended space is not confirmed, as Vas and Andersen [29] showed that animal density has no effect on resting behaviour in groups of six pregnant hornless goats, if space per animal changes between 1 m^2^ and 2 m^2^ or 3 m^2^. However, Andersen and Bøe [28] recommended that even if the organization of lying space had only a little impact on lying behaviour, agonistic interactions decreased. However, it should be taken into account that larger groups and individual needs, especially of mixed-horned herds and their impact on lying behaviour, have to be analysed as well. More feeding places might have a positive effect, as Loretz et al. [8] showed that horned goats need more space while feeding. The new AFS might structure the stable so that it is more comfortable, even for low-ranking goats, which may have more chances to obtain access to feed. This can be confirmed by Aschwanden et al. [30], who compared resting and feeding behaviour of goats with access to elevated feeding places and without, showing that elevated feeding has a positive effect especially for low-ranked and hornless goats. The positioning and the height of the new AFS seem to have an effect on lying behaviour. This can be supported by Neave et al. [13], who found out that the height of a feeding place affects the number of visits and displacements. Fewer displacements may result in less interruptions of lying goats. Additionally, the interactions at the AFS have to be analysed. Aschwanden et al. [30] showed that when elevated feeding places were available, high-ranking goats received more displacements at the feeding place and they initiated less, while on the contrary low-ranking goats received less and initiated more. This could be due to a better chance for low-ranking goats to escape. In addition to the height, the new AFS allows goats to leave in a forward direction if another goat comes from behind. This optimized AFS could have caused an adjustment in social behaviour between low-ranked and high-ranked and between horned and hornless goats (as it is well known that dominance is correlated to horn status [5]), which results in equalized lying behaviour (LBD and FLB).

## 5. Conclusions

To detect lying behaviour in goats, accelerometer technology is a practicable method with precise results, but more analyses need to be conducted to ascertain the ideal measuring interval in dairy goats. No difference was detected in lying times comparing old and new AFS, which might be traced back to several other factors influencing lying behaviour in dairy goats. However, significant differences in lying behaviour (LBD and FLB) of horned and hornless goats were found with the old AFS but not with the new one. The optimized feeding system might be a reason for reduced stress, resulting in an adjustment of lying behaviour of horned and hornless goats. Further analyses are necessary in order to evaluate lying behaviour and interactions between goats in connection with the optimized feeding system. 

## Figures and Tables

**Table 1:** Description of the different measurement periods with number of goats, age of the goats (mean ± SD) and horn status.

	April 2019	June 2019	October/November 2019	March/April 2020	June 2020	October 2020
Measurement period	04.04–11.04.	08.06–14.06.	28.10–03.11.	28.03–03.04.	13.06–19.06.	03.10–09.10.
Number of goats in the herd (n)	76 goats	72 goats	64 goats	72 goats	67 goats	65 goats
Horn status proportional in the herd	41% horned 59% hornless	42% horned 58% hornless	44% horned 56% hornless	56% horned 44% hornless	58% horned 42% hornless	57% horned 43% hornless
Number of goats per measurement (n)	19 goats	16 goats	19 goats	20 goats	20 goats	20 goats
Mean age of the evaluated goats (a)	5.28 ± 3.82	4.28 ± 2.7	4.68 ± 2.71	5.21 ± 2.37	4.3 ± 3.01	4.35 ± 3.31
Horn status of evaluated goats	11 horned 8 hornless	8 horned 8 hornless	8 horned 11 hornless	11 horned 9 hornless	13 horned 7 hornless	12 horned 8 hornless
Mean age of the evaluated goats by horn status (a)	horned: 3.09 ± 3.08 hornless: 7.75 ± 3.15	horned: 2.63 ± 1.92 hornless: 5.88 ± 2.53	horned: 2.75 ± 1.04 hornless: 6.09 ± 2.70	horned: 3.36 ± 1.12 hornless: 7.11 ± 1.90	horned: 3.00 ± 1.87 hornless: 6.71 ± 3.35	horned: 2.83 ± 2.08 hornless: 6.63 ± 3.62

Explanation: (n) = counted number, (a) = year.

**Table 2:** Results of the paired Tukey’s *t*-test (with estimated means and standard error of estimation) divided into horned and hornless goats for mean duration of lying per day (LT), mean lying boat duration per day (LBD) and mean frequency of lying boats per day (FLB).

Month	LT [h/d]		LBD [h]		FLB [n/d]	
	horned	hornless	horned	hornless	horned	hornless
April 19	12.4 ± 0.44	11.4 ± 0.51	0.51 ± 0.03 ^a^	0.66 ± 0.03 ^b^	25.7 ± 1.46 ^a^	17.4 ± 1.71 ^b^
June 19	10.8 ± 0.51	11.2 ± 0.51	0.38 ± 0.03 ^a^	0.47 ± 0.03 ^b^	29.6 ± 1.71 ^a^	24.4 ± 1.71 ^b^
October 19	11.4 ± 0.51	12.0 ± 0.44	0.52 ± 0.03	0.52 ± 0.03	23.1 ± 1.71	24.3 ± 1.46
Mean 2019	11.5 ± 0.28	11.6 ± 0.28	0.47 ± 0.02 ^a^	0.55 ± 0.02 ^b^	26.1 ± 0.94 ^a^	22.0 ± 0.94 ^b^
April 20	12.3 ± 0.44	12.7 ± 0.48	0.55 ± 0.03	0.57 ± 0.03	22.6 ± 1.46	22.3 ± 1.61
June 20	12.0 ± 0.40	11.5 ± 0.55	0.43 ± 0.02	0.43 ± 0.03	29.0 ± 1.34	27.3 ± 1.83
October 20	11.4 ± 0.42	11.4 ± 0.51	0.52 ± 0.02	0.54 ± 0.03	23.0 ± 1.40	22.0 ± 1.71
Mean 2020	11.9 ± 0.24	11.8 ± 0.30	0.50 ± 0.01	0.51 ± 0.02	24.9 ± 0.81	23.9 ± 0.99

Different superscript letters describe significant differences (*p* < 0.05) within a row belonging to one variable.

## Data Availability

Data sharing not applicable.
